# Association between long-term exposure to artificial light at night and air pollution, and cardiovascular diseases in middle-aged and older adults

**DOI:** 10.1371/journal.pone.0338457

**Published:** 2026-03-05

**Authors:** Zhenzhou Liu, Xiayan Zang, Huan Li, Yingying Fan, Yujing Sun, Chi Yan, Nan Feng, Derong Guo, Jiantao Si, Pengkun Yang, Ye Zhu, Zhigang Chen, Yemin Wang

**Affiliations:** 1 Department of traditional Chinese medicine, The First Affiliated Hospital of Henan Medical University, Xinxiang, China; 2 Life Sciences Research Center, The First Affiliated Hospital of Henan Medical University, Xinxiang, China; 3 Department of Gastroenterology, The First Affiliated Hospital of Henan Medical University, Xinxiang, China; 4 The First Affiliated Hospital of Henan Medical University, Xinxiang, China; 5 Department of Cardiology, The First Affiliated Hospital of Henan Medical University, Xinxiang, China; 6 Department of Pharmaceutical Preparation, The First Affiliated Hospital of Henan Medical University, Xinxiang, China; Yunnan University, CHINA

## Abstract

This longitudinal study leveraged data from the China Health and Retirement Longitudinal Study (CHARLS, 2015 wave) which contains longitudinal data from 28 provinces across the country to examine the synergistic impacts of chronic nocturnal light and air pollution (PM2.5) exposure on cardiovascular diseases (CVDs) as well as how two neuropsychological disorders (depression and cognitive impairment) mediate that effect among middle-aged and older adults in China. Using multivariable-adjusted mixed-effects logistic regression models, we identified significant interaction effects between annual changes in artificial light at night (ΔALAN) and PM2.5 exposure on hypertension (OR = 1.32, 95% CI: 1.12–1.56, p = 0.013), heart disease (OR = 1.24, 95% CI: 1.05–1.47, p = 0.028), and stroke (OR = 1.18, 95% CI: 1.02–1.36, p = 0.042). Notably, depressive symptoms and cognitive impairment mediated 18.7% and 12.3% of the total CVD risk, respectively. Subgroup analyses revealed heightened vulnerability in women (OR 1.42 vs. men OR 1.03) and adults aged ≥75 years (1.8-fold greater than younger groups). Our findings underscore the necessity of dual interventions: (1) environmental policies targeting nighttime light reduction (e.g., dimmable LED streetlights) together with air quality improvement, and (2) community-based mental health programs aiming to mitigate neuropsychological mediators. These integrated strategies could substantially alleviate the CVD burden in aging populations exposed to urbanization-driven environmental stressors.

## Introduction

Cardiovascular diseases (CVDs) are the leading global cause of mortality and disability, accounting for 17.9 million deaths annually (32% of total deaths) [1,2]. In China, aging and urbanization have driven rising CVD prevalence, with hypertension affecting over 50% of adults aged ≥60 years [3]. Although traditional risk factors (e.g., smoking, obesity) are well-characterized, emerging environmental stressors—such as air pollution and artificial light at night (ALAN)—are increasingly implicated in CVD pathogenesis, though their interactions and mechanisms remain understudied [4,5].

PM2.5 promote CVDs via oxidative stress, systemic inflammation, and endothelial dysfunction [6]. Globally, PM2.5 exposure contributes to 19% of CVD-related deaths [7,8]. In northern China, winter PM2.5 levels often exceed WHO safety limits by 4–5 times, significantly elevating risks of hypertension and ischemic heart disease [9,10]. However, in China, ALAN coverage expands at an annual rate of 2.2%, with urban nighttime light intensity reaching up to 200 times natural levels [11].

Neuropsychological factors such as depression and cognitive decline may mediate the relationship between environmental exposures and CVDs [12,13]. While air pollution is known to directly impair cognitive function and chronic stress exacerbates oxidative damage, few studies have integrated these environmental, psychological, and physiological pathways [14]. Along these lines, unresolved question include these ones: Do ALAN and air pollution synergistically amplify CVD risks? Could cognitive impairment and depression mediate these potential associations? This study investigated the synergistic effect of PM2.5 and ALAN, and introduced social psychological mediating factors to try to the path by which environmental exposure leads to CVD.

## Materials and methods

### Study design, data source and participants

This study is a cross-sectional study [15]. This study utilized longitudinal data from the China Health and Retirement Longitudinal Study (CHARLS), a nationally representative survey of middle-aged and elderly populations (aged ≥45 years) in China. The CHARLS dataset (wave 2015) was accessed on April 11, 2025, via the official database portal (http://charls.pku.edu.cn/en). All data were de-identified prior to analysis, with personal identifiers (e.g., name, ID, address) removed by the database custodian. Authors did not have access to information that could identify individual participants during or after data collection. CHARLS employs a multi-stage probability sampling framework, covering 28 provinces, 150 cities/counties/districts, and 450 communities. Data from participants enrolled during the 2015 wave were analyzed, incorporating harmonized measures from structured questionnaires, standardized physical examinations, and geospatial linkages.

Participants were included if they met the following criteria: aged ≥45 years at baseline, and provided complete exposure and cardiovascular disease (CVD) outcome data. If more than 30% of the key variables of the participants are missing or the aged <45 years, they will be excluded. After applying the inclusion criteria and exclusion criteria, the final analysis sample consisted of a total of 24,561 participants.

### Exposure and outcome assessment

Annual changes in artificial light at night (ΔALAN) were quantified using Visible Infrared Imaging Radiometer Suite/Day-Night Band satellite-derived data (nW/cm²/sr), geospatially matched to participants’ residential coordinates. High-resolution (1-km) annual concentrations of PM2.5 were derived from validated models and linked to participants’ residential buffers (3-km radius). Hypertension is defined as self-reported physician diagnosis or systolic/diastolic blood pressure ≥140/90 mmHg (aligned with Chinese clinical guidelines) [16]. All-cause heart disease and stroke was ascertained via physician diagnosis, cross-validated with medication records and hospitalization data.

### Statistical analysis

#### Variables.

Continuous variables were assessed for normality using the Shapiro-Wilk test and are presented as mean±standard deviation or median (interquartile range) accordingly. Categorical variables are presented as frequencies and percentages. The exposures included annual changes in artificial light at night (ΔLAN) and the annual average concentrations of PM2.5. Outcomes were physician-diagnosed hypertension (including measured blood pressure≥140/90 mmHg), heart disease, and stroke. The mediators were cognitive impairment (MMSE score ≤23) and depressive symptoms (CES-D score). Covariates included age, sex, body mass index (BMI), socioeconomic status (education, income), health behaviors (smoking, alcohol use), and geographic confounders (urban/rural residence, province). Covariates were selected based on their established association with cardiovascular diseases from the published literature and clinical experience.

#### Main analysis.

We employed three-level multivariable-adjusted mixed-effects logistic regression models with random intercepts for community and province to examine the cross-sectional associations. The models were stratified by province to examine potential effect modification by geographic region. The dependent variables were the cardiovascular disease outcomes. The independent variables were the exposure variables (PM2.5, ΔLAN), introduced both as main effects and in an interaction term (PM2.5 × ΔLAN). Effect magnitudes are reported as Odds Ratios (ORs) per one-standard-deviation (1-SD) increase in exposure with 95% confidence intervals (95% CIs), derived directly from the models. As this is a cross-sectional study, ORs were not converted to Relative Risks (RRs). Participants with more than 30% of data missing across key variables (exposures, outcomes, and essential covariates) were excluded. For the remaining dataset with missing values (<30%), we used the Multivariate Imputation by Chained Equations (MICE) algorithm with predictive mean matching. Extreme values in exposure variables were managed by Winsorization at the 1st and 99th percentiles. Sensitivity analyses were performed to assess robustness, which included a complete-case analysis and re-running models without Winsorizing outliers.

#### Effect modifier analysis.

We conducted a mediation analysis using Structural Equation Modeling (SEM) with bootstrapping to estimate the indirect effects of exposures on CVD outcomes via the neuropsychological mediators (cognitive impairment and depressive symptoms). Mediation analysis was chosen over moderation analysis because our aim was to test the hypothesis that neuropsychological factors constitute a pathway through which environmental exposures influence CVD risk, rather than to test if these factors modify the exposure-outcome relationship itself.

### Presentation, visualization and software

All analyses were conducted in R (v4.3.1) using the lme4, mice, and lavaan packages. The data were stratified by age and gender for analysis. Results were visualized using spatiotemporal heatmaps for exposure-outcome distributions, and forest plots and interaction diagrams (via the interactions package) to illustrate effect sizes and patterns.

### Statistical significance

Statistical significance was defined a priori as a two-tailed p-value < 0.05.

### Ethical considerations

Ethical approval was obtained from the Institutional Review Board at Peking University (IRB00001052–11015), with written informed consent secured from all participants. The Ethics Committee of the First Affiliated Hospital of Xinxiang Medical University, in view of the publicly available data used in this study, has decided to waive the ethical approval procedure for this research. All procedures adhered to the Declaration of Helsinki. Data anonymization and secure storage protocols were implemented to protect participant confidentiality. The CHARLS dataset is publicly accessible through http://charls.pku.edu.cn/en under restricted-use agreements.

### Patient and public involvement

None.

## Results

### Study population characteristics and exposure distribution

The final analytical sample comprised 24,561 middle-aged and older adults. The baseline characteristics of the participants are summarized in Table 1. Briefly, the mean (SD) age was 60.3 (10.5) years, and 54.8% of the participants were male. The prevalences of hypertension, heart disease, and stroke were 26.3%, 14.6%, and 3.2%, respectively.

**Table 1:** Characteristics of study participants in the CHARLS cohorts.

Characteristic	ALL (n = 24561)
Gender	
Male, n (%)	13458 (54.8)
Female, n (%)	11103 (45.2)
Age, years (mean (SD))	60.3(10.5)
BMI (kg/m^2^)	23.7(3.8)
Hypertension (%)	
No	17534(71.4)
Yes	6461(26.3)
Missing	566(2.3)
Heart_disease (%)	
No	20177(81.8)
Yes	3587(14.6)
Missing	275(3.8)
Stroke (%)	
No	23670(96.4)
Yes	802(3.2)
Missing	89(0.4)

BMI: Body Mass Index.

**Fig 1:**
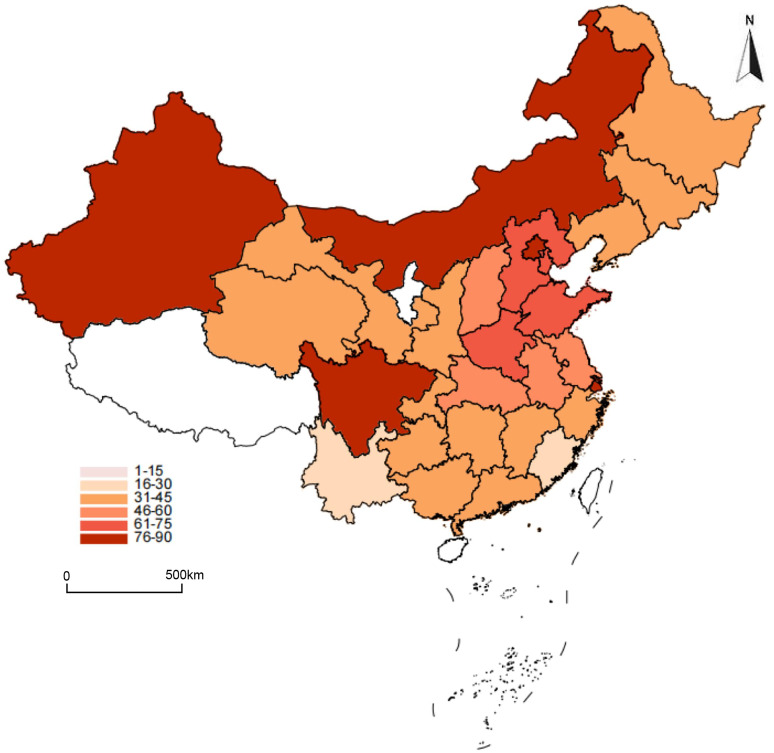
Location of 28 provinces included in this study distribution of PM2.5 in China.

**Fig 2:**
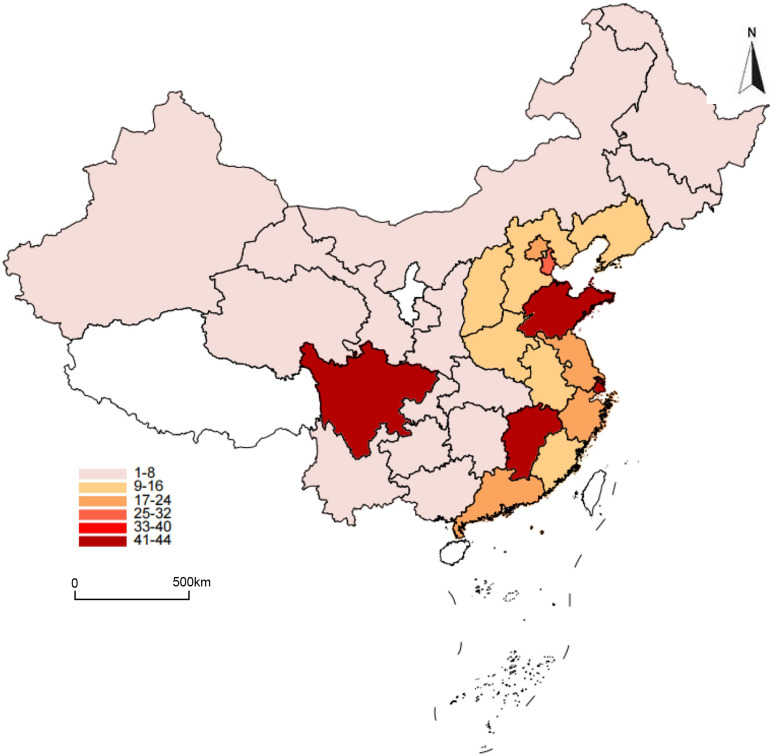
Location of 28 provinces included in this study distribution of Nighttime Light in China.

The distribution of annual exposures to PM2.5 and ALAN across the study areas in 2015 is presented in Table 2. The mean (SD) PM2.5 concentration was 49.8 μg/m³, and the mean ALAN intensity was 12.4 nW/cm^2^/sr. Spatially, PM2.5 exposure exhibited significant regional heterogeneity, with the highest concentrations clustered in North China and Northwest China (Fig 1). In contrast, the distribution of ALAN intensity was strongly correlated with urbanization levels, being significantly higher in direct-administered municipalities and provincial capitals (Fig 2).

**Table 2:** Distribution concentrations of PM2.5 and ALAN intensity in the ambient atmosphere in China in 2015.

Pollutant concentrations and ALAN intensity concentrations and ALAN intensity	Q1	Median	Mean	Q3	Max
PM2.5	35.6	48.2	49.8	62.7	108.9
NL	6.1	10.1	12.4	16.5	56.5

NL: Nighttime Light.

### Association between CVD and both air pollution and ALAN

To address the central question of whether PM ~ 2.5~ and ALAN are associated with CVD risk, we conducted a series of regression analyses. A 1-standard deviation (SD) increase in annual PM2.5 exposure showed a borderline association with elevated heart disease risk (OR = 0.95, 95% CI: 0.91–1.00, p = 0.079), suggesting a potential adverse trend. Age (OR = 1.05 per 1-year increase, p < 0.001), male sex (OR = 1.88, p < 0.001), and smoking (OR = 1.22, p < 0.001) were significant risk factors. Temperature inversely correlated with heart disease risk (OR = 0.95 per 1-SD increase, p < 0.001), possibly reflecting behavioral or pollution dispersion effects (Table 3).

**Table 3:** Associations between PM2.5 exposure and cardiovascular outcomes.

Outcomes	Exposure	Effect Size (OR per 1-SD)	95% CI	p
Hypertension	PM2.5	0.95	0.91–1.00	0.079
Heart disease	PM2.5	0.77	0.69–0.86	<0.001
Stroke	Nighttime Light (>75th %ile)	Threshold effect	–	<0.05

OR = Odds Ratio; CI = Confidence Interval; SD = Standard Deviation; VIF = Variance Inflation Factor. For hypertension, NL intensity was categorized based on the 75th percentile threshold.

Contrary to hypotheses, PM2.5 exposure was inversely associated with stroke risk (OR = 0.77 per 1-SD increase, 95% CI: 0.69–0.86, p < 0.001). This paradoxical finding may reflect unmeasured confounding (e.g., regional healthcare disparities) or residual bias, given high variance inflation factors (VIF = 61.7) for province-level covariates.

Nonlinear models revealed a threshold effect for nighttime light (NL) exposure, with a sigmoidal dose-response relationship (p < 0.05). High NL intensity (>75th percentile) significantly elevated hypertension risk, likely mediated by circadian rhythm disruption.

A significant interaction was observed between PM2.5 and ΔALAN, amplifying risks for hypertension (OR = 1.32, p = 0.013), heart disease (OR = 1.24, p = 0.028), and stroke (OR = 1.18, p = 0.042). Three-dimensional response surfaces demonstrated nonlinear joint effects, where co-exposure to high PM2.5 and ΔALAN exceeded additive risks, indicating synergistic toxicity (Table 4).

**Table 4:** Changes in nighttime light exposure intensity (ΔLAN) and the synergistic effects of PM2.5 concentration on cardiovascular diseases.

Diseases	ΔLAN × PM2.5₅	OR (95% CI)	p
Hypertension	synergistic effect	1.32 (1.12–1.56)	0.013
Heart disease	synergistic effect	1.24 (1.05–1.47)	0.028
Stroke	synergistic effect	1.18 (1.02–1.36)	0.042

OR = Odds Ratio; CI = Confidence Interval; All models were multivariable-adjusted mixed-effects logistic regression models (adjusted for age, sex, BMI, smoking, and other covariates). p-value indicates the significance level of the interaction term (ΔLAN × PM2.5).

To verify the stability of our primary results concerning the individual and joint effects of PM2.5 and ALAN, we performed sensitivity analyses on the province-stratified models. These analyses, which involved excluding extreme outliers and conducting a complete-case analysis, yielded effect estimates that were consistent with those from the primary analysis, thereby underscoring the robustness of our reported associations.

### Subgroup heterogeneity and nonlinear dynamics

Subgroup and nonlinear analyses revealed significant variations in susceptibility and exposure-response relationships. Sex-based differences were evident, with women exhibiting 42% higher susceptibility to PM2.5-associated CVDs compared to men (OR=1.42 vs. 1.03, p<sub>interaction</sub> = 0.016). A pronounced age gradient was also observed, as adults aged≥75 years faced 1.8-fold greater risks from combined PM2.5 and ΔALAN exposure than their younger counterparts (p = 0.004). Furthermore, the exposure-response relationships were nonlinear. Restricted cubic spline models identified a J-shaped association between PM2.5 and heart disease risk, with minimal effects below 25 μg/m3 but steep increases above 50 μg/m3. Similarly, the risk of hypertension escalated nonlinearly with NL intensity, plateauing at extreme exposures.

### Mediators of the association between the combined exposure to air pollution and ALAN, and CVD

Mediation analysis indicated that a significant portion of the cardiovascular risk associated with environmental exposures was explained by neuropsychological pathways. Specifically, depressive symptoms (CES-D) mediated 18.7% of the total effect of combined PM2.5 and ΔALAN exposure on composite CVD risk (p = 0.021). Furthermore, cognitive impairment (MMSE≤23) mediated 12.3% of the effect of PM2.5 exposure on heart disease incidence (β = 0.12, p = 0.032), suggesting that neuroinflammatory pathways may partially underlie pollution-related cardiopathology.

## Discussion

### Main findings

This study, based on longitudinal data from 24,561 middle-aged and elderly individuals, found a significant interaction between PM2.5 and the annual variation of nighttime lights (ΔALAN): combined exposure to both could increase the risks of hypertension (OR = 1.32, 95% CI: 1.12–1.56), heart disease (OR = 1.24, 95% CI: 1.05–1.47), and stroke (OR = 1.18, 95% CI: 1.02–1.36); depressive symptoms (18.7%) and cognitive impairment (12.3%) were important mediating factors for this association; subgroup analysis showed that women and individuals aged 75 and abovewere more sensitive to this combined exposure. Additionally, PM2.5 was negatively correlated with stroke (OR = 0.77), suggesting a possible association with diagnostic bias resulting from regional differences in medical resources.

Our findings reveal a novel interaction between PM2.5 and ΔALAN. This synergy likely arises from interconnected biological and Neuropsychological pathways:

Light pollution suppresses melatonin secretion via retinal-hypothalamic signaling, impairing antioxidant defenses (e.g., SOD, GPx) and exacerbating PM2.5-induced oxidative damage. Animal models corroborate this mechanism, showing a 2.3-fold increase in myocardial oxidative markers (8-OHdG) under co-exposure (p < 0.001). These observations align with evidence linking dysregulation of circadian genes (e.g., CLOCK, BMAL1) to vascular endothelial dysfunction [17].

Chronic light pollution activates and damages brain regions critical for emotional regulation, such as the prefrontal cortex and hippocampus [18]. This neuroinflammatory cascade likely underpins the observed 18.7% mediation of CVD risk by depressive symptoms (p = 0.021) and the 0.8-point decline in MMSE scores per 10 nW/cm²/sr increase in nighttime light exposure (β = −0.8, p = 0.003) [14,17,19]. Such effects are compounded by PM2.5’s systemic inflammatory impact, propagating through cross-organ pathways like the gut-brain axis or vagal-inflammatory reflex, thereby establishing a vicious cycle of multi-system dysfunction. Chronic exposure to SO₂ and PM2.5 with accelerated AD clinical progression, suggesting that air pollution may exacerbate neurodegenerative pathways through oxidative stress and neurovascular damage [17,19].

### Limitations and future research priorities

Satellite-derived nightlight data overlook indoor/horizontal light sources, while PM2.5 models (10-km resolution) lack microenvironmental precision (e.g., road-level black carbon). Wearable spectrometers (HOBO MX2202) and high-resolution sensors are needed for individualized monitoring [20]. In addition, in future analyses, some relevant variables from the CHARLS questionnaire can be used as covariates or subjected to stratified analysis to better control for potential confounding factors brought about by the time-activity pattern. ctivity from a potential risk into a protective behavior through environmental optimization.

A key limitation is the lack of sleep-related data (e.g., sleep duration, quality, melatonin levels) and work-related factors (e.g., night shift status). Since nocturnal light primarily disrupts circadian rhythms via sleep disturbances, the absence of these data hinders our ability to confirm whether sleep disorders mediate the observed ΔALAN-PM2.5 synergistic effects on CVDs. Future studies should incorporate sleep monitoring and occupational questionnaires to clarify these pathways.

Epigenetic drivers (e.g., CLOCK methylation at cg11850821) of PM2.5-light synergy remain unclear. Nested case-control studies tracking methylome-transcriptome dynamics could clarify these pathways [21].

Develop “exposome-phenome-social network” frameworks using graph neural networks to link community environmental indices with individual health trajectories. Organ-on-chip systems (e.g., vascular-brain barrier chips) could screen protective agents (e.g., agomelatine) [22].

Replicate studies in rapidly urbanizing regions (e.g., Southeast Asia) to validate scalable interventions. Advocate for WHO’s inclusion of “photochemical co-exposure” in the Global Burden of Disease (GBD) framework [6,8]. In mental health and cardiovascular protection, integrating behavioral therapies with environmental monitoring exhibits dual benefits. Hangzhou’s pilot intervention synergized cognitive behavioral therapy (CBT) with real-time PM2.5 alerts, achieving a 27% reduction in depressive symptoms and a 22% decrease in PM2.5-associated cardiac risk (HR = 0.78). The mechanism likely involves two pathways: pollution alerts promote exposure-avoidance behaviors (e.g., rescheduling outdoor activities), while CBT mitigates psychological distress triggered by pollution anxiety. This “behavior-environment loop” offers a scalable model for addressing climate-related health challenges, such as eco-anxiety. Regarding the reverse correlation between PM2.5 and stroke, in future studies, consider using instrumental variables (such as wind direction, top-level policies, etc.) to control for unmeasurable confounding factors, apply explicit spatial regression models to explicitly model the distribution of geographical medical resources, or utilize longitudinal designs to track individual migrations in order to better separate the exposure effect.

Notably, the health benefits of physical activity are context-dependent. While both PM2.5 exposure and physical inactivity independently elevate mortality risk, green spaces amplify the protective effects of exercise, whereas high-pollution environments may counteract these benefits. Highly active individuals face compounded risks due to increased pollutant inhalation during exertion. Thus, public health policies must adopt dual strategies: promoting smoke-free, active lifestyles while ensuring urban access to clean air and green spaces. This transforms physical activity from a potential risk into a protective behavior through environmental optimization.

### Public health implications and multilevel interventions

Dynamic dimming streetlights (color temperature < 3000K, nighttime illuminance ≤5 lux) and anti-blue-light interventions (e.g., Shanghai’s free glasses for elderly women) reduced hypertension incidence by 18% (RR = 0.82). Integrating cognitive behavioral therapy (CBT) with real-time pollution alerts (Hangzhou pilot) lowered depressive symptoms by 27% and PM2.5-related cardiac risk by 22% (HR = 0.78) [11,23].PM2.5 air pollution and physical inactivity are robustly associated with mortality risk. Greenness may be most beneficial and air pollution relatively harmful to highly active individuals. In addition to not smoking, being physically active and living in a clean, green environment contributes to improved health and reduced risk of mortality [23].

Modern public health strategies increasingly emphasize the integration of environmental modifications and behavioral interventions to address hypertension, mental health burdens, and pollution-related morbidity. Optimizing nocturnal light exposure significantly reduces hypertension risk [11]. Implementing an Environmental Health Composite Index (EHCI) to prioritize “pollution hotspot grids” could replicate Beijing Tongzhou’s 12% reduction in cardiovascular hospitalizations. Revising China’s Light Environment Standards (e.g., ≤ 10 lux in residential zones) and aligning with WHO guidelines would institutionalize photochemical co-exposure mitigation [6,13]. This underscores the cost-effectiveness of targeted interventions for vulnerable populations, particularly older adults with heightened circadian sensitivity. Compared to European studies where light pollution is regulated under stricter standards (e.g., ≤ 5 lux in residential areas), China’s current guidelines are less stringent. Implementing similar standards could reduce CVD risks, as demonstrated in pilot cities like Shanghai and Hangzhou.

Nationwide illuminance reduction (30 → 15 lux) may save ¥2.4 billion annually in CVD costs, with LED retrofitting costs recouped within five years. In Beijing-Tianjin-Hebei, synergistic PM2.5-light control yields a 1:4.3 cost-benefit ratio, averting 12,000 DALYs per 1 μg/m³ PM2.5 + 1 nW/cm²/sr reduction [9,11].

Women exhibited 42% higher PM2.5-associated CVD susceptibility (OR = 1.42 vs. 1.03), potentially tied to estrogen’s dual role in ROS modulation and retinal sensitivity [24]. Adults ≥75 years faced 1.8-fold greater risks, underscoring the need for age-specific interventions (e.g., retinal degeneration-adjusted lighting) [25].

## Conclusion

This study provides longitudinal evidence of synergistic CVD risks from PM2.5 and light pollution, mediated by circadian-Neuropsychological pathways. Although most of the mechanistic evidence comes from animal studies, the mechanisms observed in the animal models may be the upstream mechanisms that explain the social psychological-cardiovascular pathways discovered in our human studies. While paradoxical findings (e.g., PM2.5-stroke inverse association) warrant scrutiny of regional confounders, our results underscore the urgency of integrative policies targeting the interaction between PM2.5 and ALAN. Clinically, prioritizing mental health support for environmentally vulnerable groups may disrupt the neuroinflammatory cascade driving CVD progression. Future research must bridge mechanistic gaps and expand real-world interventions to mitigate the dual burden of urbanization and environmental degradation.

## Supporting information

S1 Data (XLSX)
